# The Discovery of Ether Anæsthesia

**Published:** 1894-04

**Authors:** 


### THE DISCOVERY OF ETHER ANÆSTHESIA.

"My only wish about it is to be regarded as a benefactor of my race."—CRAWFORD W. LONG.

Let us not take away from the rightful owner a reputation which enriches us not, and makes him poor indeed. Let justice have its full sway. Let us endeavor to set matters straight, regardless of national jealousies or sectional strife. Let us give credit where, and to whom credit is due. What Massachusetts has for many years tried to wrest from Georgia, must be placed under a new light, the light of truth and justice.

In the first place, the discovery of anæsthesia belongs to America. It is the gift of the New World (as antisepsis is that of the Old World) to suffering humanity. With regard especially to ether anæsthesia, great injustice has been and is still being done to its true discoverer. His name has been almost forgotten, and before the altar of praise and immortality false gods have been placed for adoration, while the true one has heretofore been consigned to oblivion, unhonored and unsung. But if it is right that we should do honor to whomsoever is worthy of it, it is time for adjustment in a question of historical veracity, a question not a question, indeed, but which has been made so, unwittingly, perhaps, but unjustly.

In his masterly address before the first Pan-American Medical Congress, Dr. William Pepper refers to "the introduction of nitrous oxide (1844) and of ether (1846) into medical practice, *with which the names of Wells and of Morton are so honorably connected*" (italics are ours), and more recently still, in the last edition of his "Principles and Practice of Surgery," Dr. John Ashhurst, jr., affirms that "the first really promising experiment in the introduction of anæsthesia dates back . . . to the year

1844,'' and again records the erroneous belief that "the first surgical operation . . . done with the aid of ether was the removal of a tumor by Dr. John C. Warren, . . . in 1846."

Can it be possible that a most worthy son, a son of whom she should be proud, should fail to get the proper recognition at the very hands of his *alma mater?* But be this as it may, it is to be regretted that such eminent authorities as Pepper and Ashhurst should still continue to disseminate an error, an error proven to be such by the plain facts of history.

Whatever credit Wells and Jackson, Morton and Warren may be entitled to in the matter at issue (and we do not wish to rob them of it), it is but fair to say that the *true discoverer* of ether anæsthesia was a Southern medical man--Dr. Crawford W. Long; and the first operation, under the influence of ether, was performed in March, 1842, by the same Dr. Long.

The question as to priority in the discovery of ether anæsthesia was first agitated by the late Dr. J. Marion Sims, in 1877, and this writer published, in May of the same year, in the *Virginia Medical Monthly*, an able article, in which he concedes said priority to Dr. Long, a man whose modesty was depriving, and still continues to deprive him of his just laurels. Quite recently, that is, in the October (1893) number of the same *Virginia Medical Monthly*, and again in the February-March (1894) number of the *Tri-State Medical Journal*, Dr. Luther B. Grandy, of Atlanta, Georgia, based upon genuine documentary evidence, published exceedingly interesting papers, in which the author proves unquestionably the justice of the claims of Dr. Crawford W. Long as the true discoverer of anæsthesia by means of ether.

It has been ascertained by Dr. Grandy that Dr. Long, while still a medical student in the University of Pennsylvania (he graduated in 1839), became acquainted with the peculiar sensations produced by inhalations of sulphuric ether. Afterwards Dr. Long stated that, after he had located in Jefferson county, Georgia, he used to give the drug to his companions, for mere amusement, and from the fact that one young man who received a sprain of the ankle-joint, while under the influence of the drug, did not perceive the slightest pain until the effects of the ether had passed off, Dr. Long was "led to believe that surgical operations might be performed under ether without pain.'' According to Dr. Grandy, "in January, 1842, a patient presented, James M. Venables by name, with two small tumors on his neck, and Dr. Long proposed to him that if 'he would submit to the operation while etherized' he 'would charge nothing, or only a nominal fee for operating.' The operation was done on the 30th of March, ether being administered on a towel. The second tumor was removed on the 6th of June following. Other operations, all of minor character, were done under the influence of ether, as occasion presented. Dr. Long waited for opportunity to test his discovery in major surgery, before publishing a report of his operations. In the mean time, Wells and Jackson and Morton made their discovery, and their first opportunity to apply the practical test came in October, 1846. In the Massachusetts General Hospital, a tumor of the neck was removed by Dr. Warren, Morton administering the ether. The successful experiment was published immediately, and Morton secured a patent on his ether, under the name of *Letheon*. Thus it was that the three New England experimenters for thirty years received the credit for a discovery which had been made four years and a half before by Dr. Long. When Morton sought to obtain a bonus from Congress for his discovery, Dr. Long presented his case also, and the merits of his claim were so well recognized that the efforts of Morton were completely frustrated. The first published account of Dr. Long's operations appeared in the *Southern Medical and Surgical Journal*, December, 1849."

Dr. Grandy deserves much credit for endeavoring, with facts historical, to remove the veil that is still thrown over the eyes of a large proportion of the medical profession. If nobody nowadays dares to deny to Harvey the discovery of the circulation of the blood, nor to Jenner that of the preventive powers of vaccine matter against small-pox, no one should dare to deny to Crawford W. Long his discovery of ether anæsthesia. Let us give credit to whom credit belongs. We seem to be more prone to accept as true statements that have been proved to be false, than to give due credit, as we should, to those noble and conscientious men who have furnished us with positive discoveries, discoveries that have been and are of real service to suffering humanity.

In the face of cold facts, words are a dead issue. The honor of the discovery of ether anæsthesia, and of its first application in the performance of a painless surgical operation, belongs, therefore, to a Southern medical man—Dr. Crawford W. Long. And while we mean no disrespect to the memory of Wells, Jackson, Morton and Warren, it is but right and just to insist in that a sacred debt should be paid to the real creditor. To him, then, who in his loftiness of purpose worked for the advancement of science and the good of the race, with the only laudable ambition of becoming a public benefactor; to him who rendered a lasting service to humanity, let us bow our heads in recognition of his great discovery. Upon the venerable crown of Crawford W. Long let us place our wreath of laurel as a simple but significant token of our love and gratitude.

Crawford W. Long was not born great, nor has he had greatness thrust upon him. He achieved greatness, and no envious tongue, no fictitious power, shall rob him of his good name. Attempts may be made at dishonoring him, perhaps, but before the grandeur of the light of immortal truth, the petty darkness of injustice and envy will vanish in despair and in shame!

It is but fitting that the Legislature of Georgia shall place the statue of Crawford W. Long in the National Gallery of Statues in the city of Washington. But that is not enough. A statue of Crawford W. Long should be raised in every medical school, in every hospital, in every public institution, the world over. His memory, more sacred, indeed, than that of kings and conquerors, should be revered by every man, woman and child, while gratitude remains unsullied in the human breast. A Southerner, Crawford W. Long, belongs not only to the South, but also to the North, the East and the West of the greatest Republic of the earth; nay, more than that, he belongs to the world, for the immortal torch of his genius lights, and shall continue "to light up the ages with the splendor of his achievement."

